# Validity of Appendicitis Inflammatory Response Score in Distinguishing Perforated from Non-Perforated Appendicitis in Children

**DOI:** 10.3390/children8040309

**Published:** 2021-04-19

**Authors:** Zenon Pogorelić, Jakov Mihanović, Stipe Ninčević, Bruna Lukšić, Sara Elezović Baloević, Ozren Polašek

**Affiliations:** 1 Department of Pediatric Surgery, University Hospital of Split, 21 000 Split, Croatia; sara.elezov@gmail.com; 2 Department of Surgery, School of Medicine, University of Split, 21 000 Split, Croatia; stipenincevic@hotmail.com (S.N.); bruna.luksic@yahoo.com (B.L.); 3 Department of Surgery, Zadar General Hospital, 23 000 Zadar, Croatia; mihanovic@gmail.com; 4 Department of Health Studies, University of Zadar, 23 000 Zadar, Croatia; 5 Department of Public Health, School of Medicine, University of Split, 21 000 Split, Croatia; opolasek@gmail.com

**Keywords:** acute appendicitis, children, AIR score, complicated appendicitis, appendicitis

## Abstract

Background: This prospective observational study aimed to evaluate the validity of appendicitis inflammatory response (AIR) score in differentiating advanced (perforated) from simple (non-perforated) appendicitis in pediatric patients. Methods: A single-center prospective cross-sectional study was conducted between 1 January 2019 until 1 May 2020 including 184 pediatric patients who underwent appendectomy. Based on the intraoperative finding of advanced (*n* = 38) or simple (*n* = 146) appendicitis the patients were divided into two groups. Recipient-operator curve (ROC), with calculation of sensitivity and specificity of best cutoff and the area under the curve (AUC), were used to measure the diagnostic value and the potential for risk stratification of the AIR score, among the patients with simple or advanced acute appendicitis. Results: The median value of the AIR score in the perforated and non-perforated groups was 10 (interquartile range, IQR 9, 11), and was 7 (IQR 6, 9), respectively (*p* < 0.001). Based on the calculated value of AIR score, the patients were classified with a high precision into low, indeterminate and high risk groups for acute appendicitis (*p* < 0.001). A cutoff value of ≥9 was demonstrated to serve as a reliable indicator of perforated appendicitis with a sensitivity and a specificity of 89.5% and 71.9%, respectively (AUC = 0.80; 95% CI: 0.719–0.871; *p* < 0.001). Conclusions: Acute appendicitis can be detected with a high level of sensitivity and specificity using the AIR score. Also, the AIR score may differentiate perforated from non-perforated appendicitis in pediatric patients with a high level of accuracy.

## 1. Introduction

It is well known that acute appendicitis is one of the most common causes of right lower abdominal pain in pediatric patients, but not every pain in that area is caused by acute appendicitis [1]. Numerous conditions can simulate the clinical presentation of acute appendicitis, like acute gastroenteritis, mesenteric lymphadenitis, torsion of intra-abdominal organs, Meckel’s diverticulitis, Crohn’s disease, enterobiasis, etc [1,2,3]. Typical clinical appearance of acute appendicitis is present only in 30–40% of pediatric patients [4,5]. On the other hand, due to the appendix’s variable position in the abdominal cavity, the clinical presentation may also significantly vary [4]. The clinical appearance of acute appendicitis in very young children is not infrequently variable, and misdiagnosis in that age group of pediatric patients is very frequent, leading to an increased rate of complications such as perforation, perityphlitis abscess, diffuse peritonitis or sepsis [1,5]. Despite advances in medicine, acute appendicitis is still a diagnostic problem and a challenge for even experienced emergency physicians and pediatric surgeons. The latter is demonstrated by the high negative appendectomy rates, ranging between 10% and 20%, documented in the literature [6,7,8,9]. The aforementioned negative appendectomy rate can be significantly lowered to 2.7% with the routine use of imaging [10]. Many scoring systems for acute appendicitis have been designed in order to help in the clinical assessment of patients. The Alvarado score and Pediatric Appendicitis Score (PAS) has been the most commonly used in the pediatric population [9]. Initial trials of the Alvarado score showed a sensitivity and specificity of 76% and 72%, respectively, while PAS showed a sensitivity of 100% and a specificity of 92% [11,12]. Later studies reported that none of the scoring systems had adequate predictive values and none can be used as an exclusive standard in establishing the diagnosis of acute appendicitis in pediatric patients [9,13,14].

In 2008, Andersson et al. introduced a new scoring system, the appendicitis inflammatory response (AIR) score, which has been created to overcome shortcomings of the Alvarado score and PAS [15]. In the AIR score, a mathematical model focused on detecting perforated appendicitis has been used. Unlike previously reported scores, this one includes C-reactive protein (CRP), which has been previously reported to have high power in discriminating between simple and advanced acute appendicitis [15,16,17]. Instead of dichotomous variables used in the previously mentioned scoring systems, in the AIR score, the clinical variables are graded according to the severity of the signs and symptoms. Also, the difference from previous scoring systems is that laboratory parameters are stratified into intervals. According to the AIR score, the patients are stratified into three cohorts: low, indeterminate and high risk of acute appendicitis. Previous studies validated the AIR score and reported that the AIR score significantly outperforms the older Alvarado score [15,16,17].

This single-center prospective cross-sectional study aimed to evaluate the value of the AIR score in distinguishing simple from complicated appendicitis in children.

## 2. Materials and Methods

### 2.1. Patients

A total of 184 patients, who met the inclusion criteria and had undergone appendectomy in our pediatric surgery clinic from 1 January 2019 to 1 May 2020, were enrolled in the single-center prospective cross-sectional study. Inclusion criteria were pediatric patients aged 0 to 17 years with acute appendicitis confirmed by histopathology. The exclusion criteria were pregnant patients, patients with chronic diseases and negative appendectomy. The parents or legal guardians of the patients gave their written informed consent before surgery.

### 2.2. Study Protocol

All appendectomies were emergencies. Study protocol recorded complete medical history, demographic data (age, sex, weight, height and body mass index), clinical data (duration of symptoms, presence of pain and/or rebound tenderness in the right inferior abdominal fossa (RIF), presence of nausea or vomiting and axillary body temperature), laboratory data (white blood cell (WBC) count, polymorphonuclear leukocytes count and CRP level), data about surgery (surgical approach (open or laparoscopic approach), severity of acute appendicitis (perforated or simple appendicitis), duration of surgery and complications) and postoperative follow-up (length of stay (LOS), postoperative complications, histopathology report). AIR score was calculated based on predictive variables for all patients (Table 1). Based on the severity of the acute appendicits, the patients were allocated into one of the study groups. In the first group, the patients had uncomplicated (non-perforated) appendicitis (*n* = 146; 79%), while in the second group, the patients had advanced (perforated) appendicitis (*n* = 38; 21%). Depending on the calculated AIR score, each patient was assigned to one of three subgroups: low (0–4), indeterminate (5–8) and high probability (9–12) of acute appendicitis.

### 2.3. Clinical Diagnosis and Indication for Surgery

A pediatric surgeon initially saw each patient and decided on surgery based on physical examination, imaging and laboratory findings. An abdominal ultrasound was used in the majority of the patients while computed tomography was used selectively at the operating surgeon’s discretion. The open or laparoscopic operating technique was based on the surgeon’s personal preference. The diagnosis and severity of acute appendicitis during the surgery was established based on the macroscopic appearance of the appendix and was confirmed histologically by a pathologist.

### 2.4. Operative Technique

Both open and laparoscopic appendectomies had standardized techniques as described previously [18,19]. In the laparoscopic approach, the appendix was skeletonized using longitudinal (Ultracision, Ethicon Endo-surgery, Cincinnati, OH, USA) or torsional harmonic scalpel (Lotus, BOWA-electronic GmbH, Gomaringen, Germany) [19,20]. The base of the appendix was secured using an endoloop (Vycril Endoloop-0, Ethicon Endo-surgery, Cincinnati, OH, USA) or polymeric clips (Ligating Clips XL, Grena, Brentford, UK) [21,22,23]. Each specimen was sent for histopathological examination. Six pediatric surgeons with a minimum of 10 years of experience in pediatric surgery performed all of the surgeries.

### 2.5. Outcome Measures

The primary outcome of the study was the validity of the AIR score in diferentiating simple from advanced appendicitis in pediatric patients. The secondary outcome was the detection of predictive factors of advanced appendicitis such as age of the patient, duration of symptoms, laboratory inflammatory markers, LOS and postoperative complications.

### 2.6. Follow-Up

Minimal follow-up was 30 days after discharge. The patients were followed up at our outpatient clinic. Follow-up protocol included the assessment of pain, the general state of the patient and the detection of late postoperative complications. Outpatient visits were scheduled seven days after surgery or discharge and finally four weeks after discharge from the hospital. 

### 2.7. Statistical Analysis

Statistical analyses were conducted using SPSS 24.0 software (IBM Corp., Armonk, NY, USA). Quantitative variables with normal distribution were described by mean and standard deviation (SD). Asymmetrically distributed quantitative variables and ordinal variables were described by median and interquartile range (IQR). Absolute and relative frequencies described distribution of categorical variables. The *t*-test for independent samples assessed the significance of differences in quantitative variables between the study groups. In the case of asymmetrical distribution of quantitative variables, the Mann-Whitney U test was used. The differences in the distribution of categorical data were assessed by using a Chi-square test. In the case of low frequency of events in a particular cell, the Fisher’s exact test was applied. The scoring system’s performance characteristics were examined using the receiver operating characteristic (ROC) curve. All the tests were two-sided and the level of significance was 0.05.

## 3. Results

Out of the 184 children included in the study, 38 (21%) had complicated appendicitis. Table 2. summarizes the demographic, clinical and laboratory data. No statistically significant difference between the investigated groups was found in regards to the patients age (*p* = 0.098), gender (*p* = 0.355), weight (*p* = 0.342), height (*p* = 0.230), body mass index (*p* = 0.679), frequency of vomiting (*p* = 0.059), pain in RIF (*p* > 0.999) and rebound tenderness (*p* > 0.999). Complication rates were similar between the groups (*p* = 0.584). The investigated groups differed significantly in regards to the duration of symptoms, axillary body temperature and level of laboratory inflammatory markers. The duration of symptoms was significantly longer (50 h (IQR 36, 84)) in the group of patients with advanced appendicitis compared to the patients with simple appendicitis (24 h (IQR 16, 30)) (*p* < 0.001). Axillary body temperature was higher (38.2 °C) in the group of patients with advanced appendicitis compared to the patients with simple appendicitis (37.4 °C) (*p* < 0.001). All investigated inflammatory laboratory markers showed statistically significant differences between the study groups: WBC count, CRP level and polymorphonuclear leukocytes were significantly higher in the perforated group of the patients compared to the group of the patients with simple appendicitis (*p* < 0.001, *p* < 0.001 and *p* = 0.003, respectively).

In both groups, laparoscopy was the preferred surgical approach, more frequent in the non-perforated group (83.6% vs. 63.2%; *p* = 0.006). No intraoperative complications were reported in both groups. During the study period, one postoperative complication occurred in the perforated group (2.6%) and two in the non-perforated group (1.4%) (*p* = 0.584). The operative time was significantly longer in the perforated group (50 min (IQR, 30, 65)) compared to 30 min (IQR 20, 40) in patients with simple appendicitis (*p* < 0.001). LOS in the perforated group was 7 days (IQR 6, 8) compared to 3 days (IQR 2, 3) (*p* < 0.001) in the non-perforated group of patients (Table 3).

Analysis of predictive factors used to calculate AIR score between the investigated groups of patients is presented in Table 4. Pain in the RIF has not been found as a predictive factor for distinguishing between simple and advanced appendicitis (*p* > 0.999). Vomiting as a predictive factor approached statistical significance in distinguishing between simple and advanced appendicitis (*p* = 0.059). Other studied predictive factors of acute appendicitis include body temperature ≥38.5 °C, leukocyte level ≥15 × 10^9^/L, polymorphonuclear granulocytes ≥85%, and serum CRP ≥50 g/L proved to be excellent predictive factors and were significantly higher in the group of patients with perforated appendicitis (*p* < 0.001).

The median AIR scores in the patients with perforated and non-perforated appendicitis were 10 (IQR 9, 11) and 7 (IQR 6, 9), respectively (*p* < 0.001).

The patients with pathohistologically confirmed acute appendicitis were allocated to either low (*n* = 8), intermediate (*n* = 103), or high risk (*n* = 73) groups (Table 5). Most (86.8%) of the patients with perforated appendicitis were placed in the high risk group, while there were no wrongly placed patients in the low risk group. Furthermore, in the group of non-perforated appendicitis, the majority (67.1%) of patients were classified in the intermediate risk group, 27.4% were placed in the high risk group and only 5.5% in the low risk group. The cutoff score of 5, for differentiating low from indeterminate and high-risk groups, yielded a sensitivity, a specificity, a positive predictive value (PPV) and a negative predictive value (NPV) of 95.6%, 50%, 98.3% and 27.2%, respectively.

**Figure 1:**
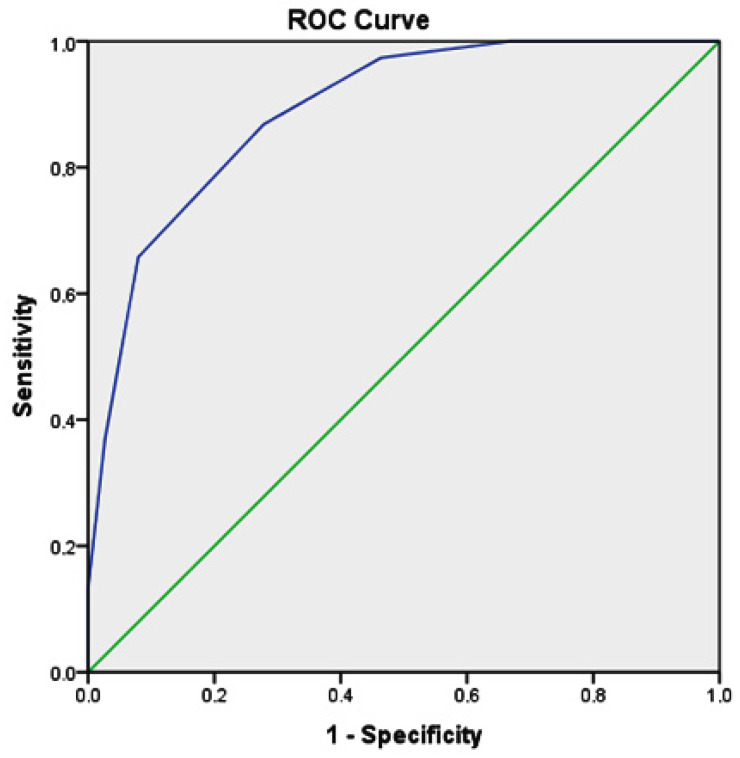
Recipient-operator curve (ROC) analysis represents the curve obtained to differentiate patients who have perforated versus those who have simple appendicitis (AUC = 0.80; 95% CI: 0.719–0.871; *p* < 0.001).

A cutoff value of ≥9 has been a good indicator of the advanced stage of inflammation. The corresponding values for the detection of perforated appendicitis alone were 89.5% for sensitivity, 71.9% for specificity, PPV 45.3%, and an NPV approaching 96.3% (AUC = 0.80; 95% CI: 0.719–0.871; *p* < 0.001) (Figure 1).

## 4. Discussion

The present study confirmed the validity of the AIR score in diagnosing acute appendicitis in pediatric patients. The score makes a significant distinction in distinguishing patients with simple and advanced appendicitis with a sensitivity of 89.5% and a specificity of 71.9%, using a cutoff value of 9. The present study also demonstrated that most (86.8%) of the patients with a perforated appendicitis were placed in the high-risk group, while there were no wrongly placed children in the low-risk group.

Numerous rating scales have been developed in recent years, based on which clinicians should decide which patients should be treated and which should be discharged for home care. The Alvarado Score and the PAS are most frequently used in children with suspicion of acute appendicitis [9,11,12,23]. However, none of them have sufficient specificity and sensitivity to be used as a reliable clinical tool. A rating scale called the AIR score has been designed to overcome the shortcomings of the Alvarado and PAS scores [17]. It uses a special mathematical model to select patients with an advanced stage of the disease. In contrast to the scales mentioned above, C-reactive protein is used as one of the variables, which has been documented in several studies as a sensitive laboratory marker in the diagnosis of acute appendicitis [13,15,17,24,25,26,27].

Clinical and laboratory variables were graded depending on the severity of symptoms and clinical signs. According to the AIR score, patients are divided into one of the following categories: high, indeterminate and low probability of acute appendicitis. This scale is originally devised for the adult population. So far, scale validations have been performed on the adult population, or rarely a mixed population that included adults and children [28,29].

Other studies showed the high value of the AIR score in the diagnosis of acute appendicitis. Macco et al. reported a similar result, they found the AUC = 0.90; 95% CI: 0.88–0.92 in their study. Comparing that result with other scoring systems, the AIR score had the highest discriminatory power, specificity and PPV [13]. This could be attributed to more objective predictive factors used in scoring, the inclusion of CRP as a variable, and the gradation of laboratory findings. Subjective factors used in other rating scales, such as nausea or anorexia, are even more challenging to determine in children who do not possess communication skills to verbalize or describe them. Scott et al. found similar results below the ROC curve (AUC = 0.84) [17]. In their study, an equal cutoff value of five was used to differentiate patients with low, indeterminate and high risk for appendicitis, with obtained 90% sensitivity and 63% specificity. The results published by Macco et al. show slightly lower sensitivity (74%) and higher specificity (87%) of AIR score in the determination of acute appendicitis [13].

The AUC to differentiate perforated from non-perforated appendicitis in this study was 0.80; 95% CI: 0.719–0.871, thus showing the excellent discriminatory ability of this score in distinguishing patients who have perforated from those who have non-perforated acute appendicitis. A cutoff value of ≥9 has been shown to be a good indicator of appendiceal perforation, with a sensitivity of 89.5% and a specificity of 71.9%. The exact cutoff value of ≥9 to differentiate patients at high-risk of acute appendicitis from patients at low and medium-risk was used in previously published studies, which showed a sensitivity of 17–23% and a specificity of 97–99%) [13,17].

The relatively high accuracy of patient stratification by risk groups shown in published studies coincides with this study’s results. Andersson et al., in their study, reported 63% of patients included in the study were classified in the low and high risk groups with a precision of 97.2%, while the remaining 37% were allocated to the indeterminate risk group. Of patients with a negative finding, 73% were classified in the low risk group and 67% of patients with complicated appendicitis were classified in the high risk group [15]. Scott et al. classified 56.5% of patients into high and low-risk groups with an accuracy of 92% in their study. Most (63.3%) of the patients without appendicitis were correctly classified as low-risk, and 41% of the patients with complicated appendicitis were classified as high risk [17].

Limitations of this research are related to the sample size and the single-center design, but the prospective design contributes to the validity of the research. Furthermore, these results should be reproduced in larger, prospective, multi-center studies, which would pave the way for the standardization of this diagnostic method and its introduction into routine clinical practice.

## 5. Conclusions

Acute appendicitis can be detected with a high level of sensitivity and specificity using the AIR score. This study has also unequivocally shown that the AIR score has a high value in distinguishing perforated from non-perforated appendicitis, which can significantly contribute to treatment decisions.

## Figures and Tables

**Table 1:** AIR score.

Predictive Factor		
Vomiting		1
Pain in the RIF of abdomen		1
Rebound tenderness/abdominal guarding	Light	1
	Medium	2
	Strong	3
Body temperature ≥38.5 °C		1
Polymorphonuclear leukocytes	<70%	0
	70–84%	1
	≥85%	2
CRP level	<10 mg/L	0
	10–49 mg/L	1
	≥50 mg/L	2
WBC count	<10 × 10^9^/L	0
	10–14.9 × 10^9^/L	1
	≥15 × 10^9^/L	2

RIF—Right inferior fossa; CRP—C reactive protein; WBC—White blood cell.

**Table 2:** Demographic characteristics, clinical and laboratory data of patients.

	Group I	Group II	*p*
	Perforated Appendicitis (*n* = 38)	Non-Perforated Appendicitis (*n* = 146)	
Demographic characteristics of patients			
Age (years) median (IQR)	10 (8, 12.5)	11 (9, 14)	0.098 ^‡^
Gender *n* (%) Male Female			0.355 ^†^
	27 (71)	92 (63)	
	11 (29)	54 (37)	
Weight (kg) mean ± SD			0.342 *
	44.2 ± 19.1	47.4 ± 17.4	
Height (cm) mean ± SD			0.230 *
	150.1 ± 22.3	154.9 ± 19.7	
BMI (kg/m^2^) median (IQR)	18.9 (16.7, 22.3)	19.1 (16.2, 24.5)	0.679 ‡
Clinical data of patients			
Duration of symptoms (h) median (IQR)	50 (36, 84)	24 (16, 30)	<0.001 ‡
Body temperature (°C) mean ± SD			<0.001 *
	38.2 ± 0.8	37.4 ± 0.6	
Vomiting *n* (%)	28 (73.7)	83 (56.8)	0.059 †
Pain in RIF *n* (%)	38 (100)	146 (100)	>0.999 †
Rebound tenderness *n* (%)	38 (100)	146 (100)	>0.999 †
Laboratory data of patients			
WBC count (×10^9^/L) mean ± SD			<0.001 *
	17.5 ± 5.3	14.2 ± 4.1	
CRP level (mg/L) mean ± SD			<0.001 *
	111.6 ± 81.3	33.6 ± 40.2	
Polymorphonuclear leukocytes (%) mean ± SD			0.003 *
	83.9 ± 6.9	80.1 ± 7.0	

* *t*-test; † Chi-square test; ‡ Mann-Whitney U-test; BMI—Body mass index; IQR—Interquartile range; RIF—Right inferior fossa; WBC—White blood cell; CRP—C-reactive protein; SD—Standard deviation.

**Table 3:** Surgical approach, treatment outcomes, and results of the pathohistological analysis.

	Group I	Group II	*p*
	Perforated Appendicitis (*n* = 38)	Non-Perforated Appendicitis (*n* = 146)	
Surgical approach, *n* (%)			
Open appendectomy	14 (36.8)	24 (16.4)	
Laparoscopic appendectomy	24 (63.2)	122 (83.6)	0.006 *
Treatment outcomes			
Complications			
Intraoperative, *n* (%)	0	0	-
Postoperative, *n* (%)	1 (2.6)	2 (1.4)	0.584 ‡
Reoperations, *n* (%)	0	0	-
Operative time (min), median (IQR)	50 (30, 65)	30 (20, 40)	<0.001 §
LOS (days), median (IQR)	7 (6; 8)	3 (2; 3)	<0.001 §
Pathohistological analysis, *n* (%)			
Catarrhal appendicitis	0	1 (0.7)	
Phlegmonous appendicitis	0	88 (60.3)	
Gangrenous appendicitis	38 (100)	57 (39)	

* Chi-square test; ‡ Fisher’s exact test; § Mann-Whitney U-test; IQR—Interquartile range; LOS—length of hospital stay.

**Table 4:** Predictive factors analysis of the AIR score.

Predictive Factor, *n* (%)		Group I	Group II	p *
		Perforated Appendicitis (*n* = 38)	Non-Perforated Appendicitis (*n* = 146)	
Vomiting		28 (73.7)	83 (56.8)	0.059
Pain in RIF		38 (100)	146 (100)	>0.999
Axillary temperature ≥38.5 °C		18 (47.4)	16 (11)	<0.001
Polymorphonuclear leukocytes	<70%	2 (5.3)	10 (6.8)	<0.001
	70–84%	10 (26.3)	92 (63)	
	≥85%	26 (68.4)	44 (30.2)	
CRP level	<10 mg/L	1 (2.6)	45 (30.8)	<0.001
	10–49 mg/L	9 (23.7)	68 (46.6)	
	≥50 mg/L	28 (73.7)	33 (22.6)	
WBC count	<10 × 10^9^/L	2 (5.3)	23 (15.8)	<0.001
	10–14.9 × 10^9^/L	12 (31.6)	71 (48.6)	
	≥15 × 10^9^/L	24 (63.2)	52 (35.6)	
AIR score median (IQR)		10 (9, 11)	7 (6, 9)	<0.001 †

* Chi-square test; † Mann-Whitney U-test; RIF—Right inferior fossa; CRP—C-reactive protein; WBC—White blood cell; AIR—Appendicitis inflammatory response; IQR—Interquartile range.

**Table 5:** Patient risk stratification based on the AIR score.

*n* (%)	High Risk (Score ≥ 9)	Indeterminate Risk (Score 5–8)	Low Risk (Score < 5)
Non-perforated appendicitis	40 (27.4)	98 (67.1)	8 (5.5)
Perforated appendicitis	33 (86.8)	5 (13.2)	0

## Data Availability

The data presented in this study is available upon request of the respective author. Due to the protection of personal data, the data is not publicly available.
